# RETRACTION: Antiapoptotic and Antioxidant Properties of *Orthosiphon stamineus* Benth (Cat's Whiskers): Intervention in the Bcl-2-Mediated Apoptotic Pathway

**DOI:** 10.1155/ecam/9793087

**Published:** 2025-11-25

**Authors:** Evidence-Based Complementary and Alternative Medicine

RETRACTION: S. I. Abdelwahab, S. Mohan, M. M. Elhassan, et al., “Antiapoptotic and Antioxidant Properties of *Orthosiphon stamineus* Benth (Cat's Whiskers): Intervention in the Bcl-2-Mediated Apoptotic Pathway,” *Evidence-Based Complementary and Alternative Medicine* 2010, no. 1 (2010): https://doi.org/10.1155/2011/156765.

The above article, published online on 30 December 2010 in Wiley Online Library (https://wileyonlinelibrary.com), has been retracted by John Wiley & Sons Ltd. The retraction has been agreed after an investigation into concerns raised by *Alatospora acuminata* via PubPeer [1].

Specifically, unexpected similarities were detected between the western blot bands in Figure 5 and the bands in Figure 3 of [2], which received different treatments.

Multiple attempts by the journal to contact the authors have not resulted in a reply. As such, the conclusions reported in the article are not considered reliable.
